# Impact of Cocoa Consumption on Inflammation Processes—A Critical Review of Randomized Controlled Trials

**DOI:** 10.3390/nu8060321

**Published:** 2016-05-26

**Authors:** Sabine Ellinger, Peter Stehle

**Affiliations:** 1 Faculty of Food, Nutrition and Hospitality Sciences Hochschule Niederrhein, University of Applied Sciences, Rheydter Str. 277, Mönchengladbach 41065, Germany; 2 Department of Nutrition and Food Sciences, Nutritional Physiology, University of Bonn, Endenicher Allee 11-13, Bonn 53115, Germany; pstehle@uni-bonn.de

**Keywords:** cocoa, chocolate, inflammation, randomized controlled trials, evidence, critical review

## Abstract

Background: Cocoa flavanols have strong anti-inflammatory properties *in vitro*. If these also occur *in vivo*, cocoa consumption may contribute to the prevention or treatment of diseases mediated by chronic inflammation. This critical review judged the evidence for such effects occurring after cocoa consumption. Methods: A literature search in Medline was performed for randomized controlled trials (RCTs) that investigated the effects of cocoa consumption on inflammatory biomarkers. Results: Thirty-three RCTs were included, along with 9 bolus and 24 regular consumption studies. Acute cocoa consumption decreased adhesion molecules and 4-series leukotrienes in serum, nuclear factor κB activation in leukocytes, and the expression of CD62P and CD11b on monocytes and neutrophils. In healthy subjects and in patients with cardiovascular diseases, most regular consumption trials did not find any changes except for a decreased number of endothelial microparticles, but several cellular and humoral inflammation markers decreased in patients suffering from type 2 diabetes and impaired fasting glucose. Conclusions: Little evidence exists that consumption of cocoa-rich food may reduce inflammation, probably by lowering the activation of monocytes and neutrophils. The efficacy seems to depend on the extent of the basal inflammatory burden. Further well-designed RCTs with inflammation as the primary outcome are needed, focusing on specific markers of leukocyte activation and considering endothelial microparticles as marker of vascular inflammation.

## 1. Introduction

Chronic inflammation has been observed in several chronic degenerative diseases beyond rheumatoid arthritis, e.g., in coronary artery disease (CAD) [1], diabetes mellitus [2], age-related macular degeneration [3], Parkinson’s, and Alzheimer’s disease [4]. Chronic inflammation is a decisive risk factor for the development of vascular diseases as it plays a key role in all stages of the formation of vascular lesions and leads to endothelial dysfunction [1]. Biomarkers in serum and plasma like proinflammatory cytokines, adhesion molecules, and acute-phase reactants such as C-reactive protein (CRP) are related to increased risk of cardiovascular events [5] and diabetes-associated vascular impairment [6].

This explains the increasing interest in nutrients with anti-inflammatory properties. A growing body of evidence from *in vitro* studies suggests that nutritive flavanols can modulate the synthesis of pro- and anti-inflammatory metabolites. Flavanols have been shown to reduce the transcription and secretion of adhesion molecules and proinflammatory cytokines (interleukin-1β (IL-1β), tumor necrosis factor-α (TNF-α)) [7,8,9,10]. Moreover, flavanols increased the production of anti-inflammatory cytokines such as interleukin-4 and interleukin-5 *in vitro* [11]. Since epicatechin, catechin, and dimeric procyanidins inhibit the activation of nuclear factor κB (NFκB) *in vitro* at multiple stages [12,13], the lowered transcription of adhesion molecules and proinflammatory cytokines *in vitro* may result from changes in redox-sensitive signaling pathways like those of NFκB [14,15,16]. The inhibition of both lipoxygenases [17,18] and matrix metalloproteinases [19] observed *in vitro* may contribute to anti-inflammatory effects [20].

Cocoa products are popular foods rich in flavanols. This refers especially to dark chocolate which has the highest flavanol content among flavanol-rich foods per 100 g food [21]. Cocoa products contribute to the four major dietary flavanol sources in Europe [22]. Cocoa flavanols encompass catechin and epicatechin as monomers and procyandins as oligomers. The procyandins differ from each other by the number and kind of monomers. In cocoa, oligomers with 4→8 and 4→6 linkages are predominant [21,23]. Increasing evidence suggests that regular cocoa consumption contributes to cardiovascular health by reducing blood pressure [24,25,26,27,28], LDL-cholesterol [25,27,29,30], and insulin resistance [25,27], and by improving vascular elasticity [25,27,28]. These effects are ascribed to cocoa flavanols [25,31,32,33], especially to epicatechin [34,35,36]. In the United States, mean flavanol intake was 158 mg/day, estimated from the USDA Flavonoid Database and 24 h dietary recalls from NHANES 1999–2002 [37]. In the EPIC study, average total flavanol intake ranged from 161 mg/day (Greece) to 406 mg/day (UK), an estimation also based on 24-h recall, but considering a larger database (the USDA Flavonoid Database and Phenol-Explorer). In Europe, cocoa products contribute to 5% of total flavanol intake [38], which corresponds to an average intake of 7–19 mg/day.

A cohort study has shown that the consumption of small doses (up to 20 g) of dark chocolate was associated with low concentrations of CRP [39]. However, meta-analyses of randomized controlled trials (RCTs), which included data from five [27] and 10 [25] individual trials, respectively, did not find any changes in CRP by cocoa consumption. These meta-analyses were published in 2011 [27] and 2012 [25], respectively. Up to now, results from RCTs on biomarkers like adhesion molecules and proinflammatory cytokines, known to be involved in different phases of atherosclerosis [5,6,40], have not been assembled yet. The impact of cocoa consumption on inflammation *in vivo*, based on RCTs, is not clear. In order to evaluate the evidence of anti-inflammatory effects obtained by cocoa consumption, the RCTs’ quality should also be considered.

The aim of this review is to provide a complete overview on the effects of cocoa consumption on markers of inflammation from RCTs with consideration of the quality of the studies to judge the evidence for anti-inflammatory effects *in vivo*.

## 2. Materials and Methods

### 2.1. Literature Search

A systematic literature search was performed in Medline via PubMed for RCTs published between January 2000 and April 2016 that investigated the effects of cocoa-rich food consumption on markers of inflammation. The first search combined the keywords “cocoa” OR “cacao” OR “chocolate” with “inflammation” OR “eicosanoids” OR “adhesion molecule(s)”. The second search combined the keywords “cocoa” OR “cacao” OR “chocolate” with “cardiovascular”. The second search was less specific, but necessary to detect relevant studies that did not focus on inflammation and that were not found by the first search strategy. For the search in PubMed, two filters were applied to restrict the records by type and language to clinical trials published in English or German. In addition to the PubMed search, other sources such as meta-analyses and reviews on the effect of cocoa consumption on inflammation were examined for relevant studies.

### 2.2. Selection of Trials

The following eligibility criteria were considered:

1. Type of studies: randomized controlled trials
2. Type of interventions: supplementing foods rich in cocoa without restrictions to the kind of food used (e.g., dark chocolate, cocoa-rich drinks, cocoa-enriched foods) or the frequency of supplementation (e.g., single or repeated, daily consumption)
3. Type of controls: supplementing comparable foods lacking cocoa (e.g., white chocolate, milk, placebo drink, foods not enriched with cocoa), being low in cocoa or not providing cocoa-rich foods
4. Type of inflammatory biomarkers: humoral parameters determined in serum or plasma (e.g., CRP, soluble adhesion molecules, eicosanoids, cytokines) and cellular parameters determined in leukocytes obtained from whole blood (e.g., cellular adhesion molecules, immunological functions)

All records identified were checked for duplicates. Duplicates were removed and the remaining records were screened by title and/or abstract to exclude records that did not contribute to answering the question asked in this review. The full-text articles of the remaining records were assessed for eligibility on the basis of the criteria mentioned above.

### 2.3. Criteria for Judging the Quality of the Selected Trials

The quality of the selected RCTs was judged according to the GRADE criteria. Methodological limitations, e.g., lack of allocation concealment, lack of blinding (participants, researchers), lack of reasons for dropout, no data on compliance, industrial funding, and carryover effects in case of crossover trials were considered. This approach is helpful to assess the quality of evidence more differently by rating down the evidence in case of serious limitations [41,42]. Recording the dietary intake and imposing dietary restrictions on the consumption of other flavanol-rich foods than cocoa may exclude potential side effects and were considered as additional criteria to assess the studies’ quality.

## 3. Results

**Figure 1:**
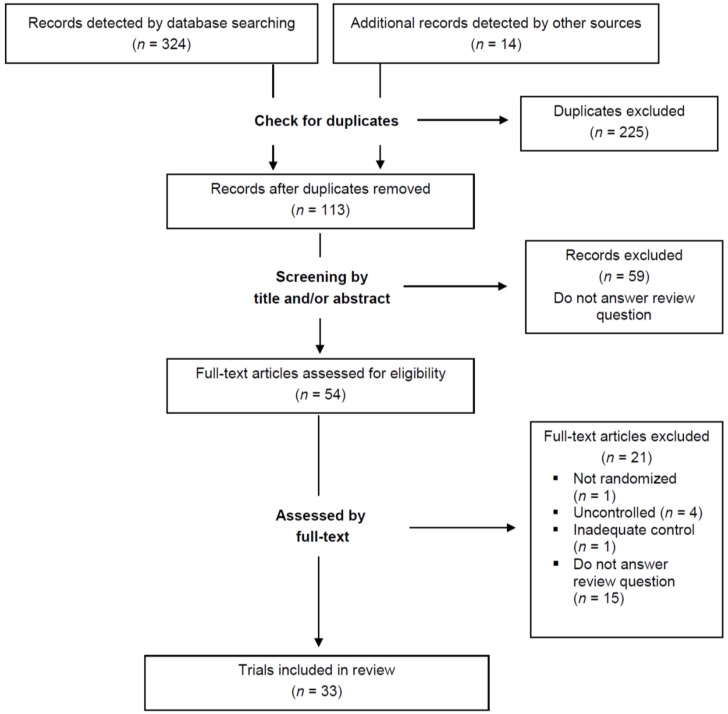
Flow diagram of study selection process.

Figure 1 provides an overview of the selection of relevant studies. In total, 338 records could be identified. After removing 225 duplicates, the remaining records (*n* = 113) were screened by title and/or by abstract. This led to the exclusion of 59 records that were considered to be irrelevant to the question addressed by this review. The remaining 54 records were checked for eligibility by the full-text article, leading to the exclusion of 21 articles. Finally, 33 RCTs were included in this review.

These 33 trials were published in 32 different articles. Nine trials investigated the effect of acute cocoa consumption [43,44,45,46,47,48,49,50] (Table 1) and 24 trials addressed the impact of regular, *i.e.*, daily, cocoa consumption on markers of inflammation. The regular consumption trials were further subdivided according to the subjects investigated: trials performed with healthy subjects [51,52,53,54,55,56,57,58,59] (Table 2), pre-/hypertensive subjects [51,60,61,62] (Table 3), and trials investigating patients suffering from type 2 diabetes, impaired glucose tolerance [61,63,64,65,66,67,68] (Table 4), and from CAD [48,69,70] (Table 5). For treatment, most trials used dark chocolate [43,45,47,48,49,50,51,52,53,58,59,61,68,69,71] or cocoa drinks rich in flavanols [44,46,50,54,55,57,59,60,62,63,64,66,67,70,72]. Chocolate or drinks low [43,44,49,55,58,60,62,63,66,70,71,72] or even free [45,46,47,48,50,51,53,54,55,56,57,59,61,64,65,66,67,68,69] from cocoa mostly served as controls. A few RCTs addressed the effect of flavanol-rich cocoa on postprandial [43,50] or hyperglycemia-induced inflammation [49,66].

### 3.1. Bolus Studies

Table 1 provides on overview on bolus studies (*n* = 9) included in this review. In healthy subjects, the consumption of cocoa-rich food decreased the concentration of leukotrienes C_4_, D_4_, and E_4_ in plasma [43], the expression of CD62P (P-selectin) on collagen-activated monocytes and neutrophils [44], and the serum concentration of intercellular adhesion molecule-1 (ICAM-1) and E-selectin [46]. A reduction in E-selectin was only observed if cocoa-rich beverages were prepared with water. A reduction in ICAM-1 was induced after ingestion of water and milk based cocoa-rich beverages, but concentrations after 6 h were comparably lower after ingestion of the water-based drink. Interestingly, the ratio of P-p65/β-actin in peripheral blood mononuclear cells (PBMC) decreased after consuming the water-based cocoa drink, but not after the milk-based cocoa drink, and even increased after consumption of pure milk. In contrast to ICAM-1, vascular cell adhesion molecule-1 (VCAM-1) did not change after any kind of drink [46]. In healthy non-smokers with prolonged exercise, consumption of 100 g of dark chocolate 2 h prior to prolonged exercise did not change IL-6 in plasma and neutrophils’ respiratory burst and degranulation when compared to the cocoa-free chocolate and fasting conditions [47]. In patients with type 2 diabetes, ingestion of 13.5 g chocolate rich in flavanols 60 min after an oral 75-g-glucose load did not change ICAM-1, E-selectin, P-selectin, and P-selectin-glycoprotein ligand 1 in serum, whereas ICAM-1 increased after ingestion of the same amount of flavanol-poor chocolate [49]. It is important to mention that the differences between pre- and post-consumption values of these four parameters were significantly lower for the flavanol-rich compared to the flavanol-poor chocolate. In type 2 diabetics, a placebo-controlled trial investigating whether a cocoa-rich drink consumed together with a high-fat fast-food style meal might affect CRP, but CRP levels did not change after either intervention, and differences between pre- and post-consumption values could not be observed [50]. In patients with peripheral artery disease, ingestion of 40 g dark chocolate reduced the concentration of NOX2-derived peptide, a marker of NADPH oxidase activity, in serum 2 h after consumption, whereas no changes occurred after consumption of milk chocolate [71]. In patients with congestive heart failure [48] and in heart transplant recipients [45], bolus consumption of 40 g dark or cocoa-free chocolate did not change CRP.

### 3.2. Studies on Regular Cocoa Consumption

#### 3.2.1. Healthy Subjects

Table 2 provides on overview of studies (*n* = 9) that investigated the impact of regular consumption of cocoa-rich products on markers of inflammation in serum or plasma in healthy subjects after an overnight fast. ICAM-1 decreased after daily consumption of dark chocolate (41 g/day) for 42 weeks [52], but not after providing 100 g/day chocolate for 15 days [51] or 400 mL/day of a cocoa-rich beverage for 28 days [57]. In the study of Ibero-Baraibar *et al.* [56], ICAM-1 decreased by consumption of ready-to-eat-meals irrespective if these were enriched with cocoa extract or not. However, these meals were part of a hypocaloric diet with an energy restriction about 15% of total energy expenditure [56]. Regular cocoa consumption did not change VCAM-1 in any study [52,56,57]. Changes in proinflammatory cytokines (IL-1β, IL-6, TNF-α) were not observed [57,58]. The concentration of chemokines in plasma (IL-8, monocyte chemoattractant protein-1) was not modulated by 4-week consumption of a cocoa drink [57]. However, interleukin-10 decreased after intervention with cocoa-rich and cocoa-free drinks, but the changes induced by the cocoa drink were significantly lower than those of the cocoa-free control [57]. In most trials that determined serum CRP concentrations, changes did not occur due to regular cocoa consumption [51,52,53,54,57,58]. Only Tzounis *et al.* found a decrease in CRP after 4-week consumption of a flavanol-rich cocoa drink, which was accompanied by significantly lower post-consumption values compared to the low-flavanol cocoa drink [55]. In three studies, results on CRP were not reported [57,58,59]. In another study [59], reduced concentrations of haptoglobin and endothelial microparticles in serum and plasma, respectively, and a lower expression of CD62L (l-selectin) on monocytes after 4-week consumption of cocoa-rich bars were observed in obese subjects, but not in similar aged subjects who were normal weight or overweight. In obese subjects, a trend towards reduced levels of endocan-1 (also called endothelial cell specific molecule-1) and E-selectin in serum after cocoa consumption could be observed, which, however, did not reach significance. At baseline, the concentrations of haptoglobin, E-selectin, and endothelial microparticles were significantly higher in obese subjects compared to normal-weight and overweight subjects. Moreover, obese participants initially had a higher expression of CD62L on monocytes than participants who were normal weight or overweight.

#### 3.2.2. Pre-/Hypertensive Subjects

The results of RCTs performed with pre-/hypertensive subjects are shown in Table 3. In patients with untreated stage I hypertension, daily consumption of 100 g dark chocolate for 15 days did not affect ICAM-1 [51] and CRP [51,61]. Consumption of flavanol-rich cocoa drinks for two [62] or six weeks [60], respectively, did not change E-selectin [60,62], P-selectin [60], ICAM-1, VCAM-1, monocyte chemoattractant protein-1 (MCP-1), IL-6, or TNF-α [62] in serum or plasma. The only significant change observed concerned VCAM-1 in the study of Wang-Polagruto *et al.* [60]. Here, VCAM-1 decreased after six weeks’ ingestion of a cocoa-rich drink and the differences between pre- and post-consumption values were higher after consuming the cocoa-rich compared to the control drink.

#### 3.2.3. Patients with Type 2 Diabetes or Impaired Glucose Tolerance

Studies with patients suffering from type 2 diabetes or impaired glucose tolerance are shown in Table 4. Daily administration of flavanol-rich chocolate (25 g [68], 45 g [65], 100 g [61]) for 5–56 days compared to white chocolate did not change CRP. Most studies providing cocoa-rich beverages observed anti-inflammatory effects: Parsaeyan *et al.* found a decrease in CRP, IL-6, and in TNF-α after six weeks [67]. Stote *et al.* [66] provided water-based beverages with different amounts of flavanols for five days to a group of obese patients who suffered from impaired glucose tolerance. Before and after intervention, an oral 75-g-glucose load was given and a significant decrease in ICAM-1, IL-6, and in CRP by ingestion of the cocoa drink was observed, dependent on the dose of flavanols ingested. Balzer *et al.* [63] also provided flavanol-rich cocoa, but did not find any changes in CRP. However, this was the only inflammatory biomarker determined in this trial. Monagas *et al.* [64], who investigated a mixed group of patients (diabetics and patients with at least three cardiovascular disease risk factors such as smoking, hypertension, hypercholesterolemia, obesity, and a family history of premature coronary disease), provided 500 mL/day of a flavanol-rich cocoa drink, based on milk, and pure milk as a control for 28 days. Cocoa consumption reduced the concentration of several adhesion molecules in plasma (ICAM-1, P-selectin) and on monocytes (very late antigen-4; VLA-4; CD49d), and the expression of CD36 and CD40 on monocytes. For these parameters, differences between pre- and post-consumption values were significantly higher after consumption of cocoa-rich cocoa compared to milk. E-selectin, VCAM-1, MCP-1, IL-6, and CRP in serum or plasma and lymphocyte function-associated antigen-1 (LFA-1; CD11a), Mac-1 (CD11b/CD18), Sialil Lewis X (SLe^x^; CD15s) on monocytes did not change. The expression of LFA-1, VLA-4, SLe^x^, and CD40 on T cells was not modulated by cocoa intervention either.

#### 3.2.4. Patients with Coronary Heart Disease

In patients suffering from CAD [69,70,72] and congestive heart failure [48] (Table 5), daily ingestion of flavanol-rich chocolate [48,69] or cocoa [70,72] for 28–42 days did not change CRP [48,69,70], P-selectin, E-selectin, ICAM-1, or VCAM-1 [69]. However, VCAM-1 was significantly higher before the consumption of flavanol-rich chocolate compared to isocaloric placebo. This difference was no longer significant after six weeks of intervention [69]. Chemotaxis of PBMC was not modulated by consumption of flavanol-rich cocoa [70]. The number of endothelial microparticles (CD144+ as well as CD31+/CD41- subpopulation) decreased significantly after ingestion of flavanol-rich cocoa drinks and reached lower concentrations after 30 days of intervention compared to the placebo drinks. At baseline, CAD patients had more CD31+/CD41- endothelial microparticles in blood than age-matched and younger healthy controls, whereas the number of CD144+ endothelial microparticles differed only between young and old subjects, but not between CAD patients and age-matched healthy controls. In contrast to endothelial microparticles, the number of platelet-derived microparticles (CD41+) was not affected by cocoa treatment [72].

### 3.3. Quality of Selected Studies

Table 6 provides an overview on qualitative criteria of the selected trials. Seventy percent of them had crossover and 30% had a parallel group design. Masking of researchers and participants was ensured in 82% and 61% of all RCTs, respectively. Information on allocation concealment was lacking in half of all studies. Dropout was clearly reported in most studies with regular cocoa consumption and also in bolus studies if patients were lost, but this did rarely occur. Forty percent of all studies were funded by industry and most trials received the cocoa products as gifts from industry (82%). In bolus studies, compliance was not reported as cocoa products were always ingested in the study center. Only 50% of regular consumption studies determined adherence to dietary treatment. Carry-over effects could be excluded in most trials with crossover design by washout periods. About 45% of all studies recorded dietary intake and 76% provided dietary restrictions to prevent confounding effects.

## 4. Discussion

To the best of our knowledge, this is the first review that critically evaluates the impact of cocoa consumption on inflammatory markers on the basis of RCTs.

After consumption of cocoa-rich food, most studies observed a decrease in inflammatory biomarkers in healthy subjects after 2–6 h (Table 1). This includes a reduction in 4-series leukotrienes [43], E-selectin, and ICAM-1 in serum/plasma [46], as well as a lowered expression of CD62P on monocytes and neutrophils [44]. The reduced activation of NFκB in PBMC, indicated by the lowered ratio of P-p65/β-actin after ingestion of a water-based cocoa drink [46], suggests that cocoa flavanols can reduce the transcription of several genes encoding for inflammatory markers like ICAM-1 and E-selectin *in vivo* [73]. In contrast to bolus consumption, regular cocoa consumption did not change the serum/plasma concentration of VCAM-1 [52,56], ICAM-1 [56,74], IL-1β, IL-6, TNF-α [57,58], IL-8, MCP-1 [57], E-selectin [59] and CRP [51,52,53,54] in healthy subjects, except for the decrease in CRP observed by Tzounis *et al.* [55]. Only McFarlin *et al.* observed a decrease in selected parameters (haptoglobin, CD62L on monocytes, endothelial microparticles) after regular cocoa consumption. This decrease was only significant in obese subjects who had initially higher values than subjects who were normal weight or overweight [59] (Table 2).

The different results from bolus and regular consumption studies in healthy subjects raises the question of why anti-inflammatory effects were detectable after acute cocoa consumption, but not in most studies after regular cocoa consumption. First of all, we have to bear in mind that epicatechin, which is claimed to be responsible for anti-inflammatory effects after cocoa intake [31], has an elimination half-time about 2 h [75]. For this reason, anti-inflammatory effects are rather unlikely to occur in blood samples obtained after an overnight fast in regular consumption studies. On the other hand, epicatechin metabolites were found in lymphoid tissues of rats after daily cocoa feeding for three weeks [76]. Consequently, an accumulation of epicatechin in human leukocytes and also in endothelial cells by regular cocoa consumption may occur. Second, if we consider that a balanced release of vasodilating/-constricting factors and pro-/antithrombotic substances prevents vascular inflammation in healthy subjects [77], the lack of anti-inflammatory effects after regular cocoa consumption in fasting blood may be simply explained by the lack of basal inflammation. This is stressed by the results of McFarlin *et al.*, who found anti-inflammatory effects after regular cocoa consumption only in obese participants with initially elevated inflammation markers compared to normal-weight and overweight subjects, but not in the later subgroups [59]. A range of proinflammatory adipokines are released from adipose tissue in obesity [78], but most RCTs on regular cocoa consumption did not investigate healthy subjects with obesity (Table 2).

In contrast to healthy subjects, most regular consumption trials with patients suffering from type 2 diabetes or impaired glucose tolerance found lower concentrations of ICAM-1 [64,66] and P-selectin in serum or plasma [64] and a reduced expression of VLA-4, CD36, and CD40 on monocytes [64]. Results on proinflammatory cytokines and on CRP are divergent (Table 4), but a reduction could be observed in two studies for proinflammatory cytokines [66,67] and in three studies for CRP [66,67,68] (Table 4). In pre-/hypertension (Table 3) and in CAD (Table 5), two studies only observed a reduction in VCAM-1 by regular cocoa consumption [60,69]. The activity of NADPH-oxidase [71] and the number of endothelial microparticles [72] decreased after acute [71] and regular consumption [72] of flavanol-rich cocoa products, respectively. However, further trials did not find any changes after acute [45,48] and regular consumption [48,51,61,62,70]. The results from different groups of participants suggest that the reaction towards cocoa consumption is different. This may be explained by the different stages of vascular inflammation. In patients with type 2 diabetes, vascular endothelial dysfunction can be partly restored by reducing inflammation [79]. In these patients, including those with impaired glucose tolerance, anti-inflammatory effects could be observed in several studies after cocoa consumption at the basal state, but also after an oral glucose challenge (Table 4). As vascular inflammation in patients with existing cardiovascular diseases is pronounced and associated with proatherogenic modifications [77], the anti-inflammatory properties of cocoa flavanols that were ingested from cocoa-rich foods might have been too weak to reduce inflammation in pre-/hypertensive subjects and in patients with existing CAD.

The anti-inflammatory effect of cocoa depends on the dose of cocoa flavanols ingested [66], but data on total flavanol intake based on HPLC analysis of individual flavanols are only provided by single studies. As the epicatechin content in cocoa products correlates strongly with the sum of catechin, epicatechin, dimer B2, dimer B5, trimer C1, and tetramer D (*r* = 0.99) [80], epicatechin intake with cocoa reflects total flavanol intake. Therefore, the dose of epicatechin, which is believed to be responsible for the anti-inflammatory effects of cocoa consumption [31], has to be considered for the interpretation of the results. However, data on epicatechin intake were not available from all studies. Apart from the dose, efficacy on inflammatory biomarkers may depend on the food matrix. A higher bioavailability can be achieved by using a liquid matrix (cocoa drink) instead of solid chocolate, by co-administration of carbohydrates (e.g., by sugar or a carbohydrate-rich meal), and by the lack of milk protein [23]. Most studies providing flavanol-rich chocolate did not find anti-inflammatory effects [45,47,48,51,58,61,65,68] except for four studies [43,49,52,69] in which, however, only single parameters changed. On the other hand, nine out of 14 RCTs providing a flavanol-rich cocoa drink observed anti-inflammatory effects in one [44,55,57,60] or even several parameters [46,64,66,67]. However, methodological differences concerning participants (age, BMI, health status, or stage of vascular inflammation), the kind of intervention (food matrix, dose of epicatechin ingested), and the biomarkers used make a comparison between studies difficult. In regular consumption trials, the duration of cocoa treatment might also be relevant if epicatechin would accumulate *in vivo*. A reduction in ICAM-1 and ICAM-3 as well as a decreased expression of several adhesion molecules on leukocytes was observed after daily consumption of 70 g dark chocolate by overweight men, irrespective of whether the chocolate was enriched with flavanols or not [81]. This suggests that an epicatechin intake >97 mg through dark chocolate does not lead to stronger anti-inflammatory effects in overweight men. If other patients or foods were used, the result might have been different. For these reasons, a minimum dose that is needed to reduce inflammation cannot be ruled out.

Furthermore, the choice of parameters has to be considered when interpreting the results. CRP did not change in most studies despite the use of highly-sensitive CRP kits (Table 1, Table 2, Table 3, Table 4 and Table 5), which are demanded for subjects with cardiovascular risk or established cardiovascular diseases [82]. In bolus studies, the lack of changes in CRP 2 h [45,48] and 6 h [50] after bolus consumption of cocoa is not surprising as the CRP’s reaction *in vivo* to stimuli such as IL-1β, IL-6, and TNF-α takes about 6 h [83]. In regular consumption trials, the lack of changes in CRP indicates that cocoa consumption does not affect systemic inflammation, and the main stimuli for CRP synthesis, IL-1β, IL-6, and TNF-α, did not change in most trials. However, due to their short half-life time *in vivo* (for TNF-α only 5 min) and their low stability in serum or plasma samples, the concentrations *in vivo* may be underestimated [84]. This is likely as several adhesion molecules whose gene expression is induced by TNF-α and IL-1β [85] decreased in serum/plasma in several studies. As shown in Table 1, Table 2, Table 3, Table 4 and Table 5, nearly 50% of all trials found a reduction in ICAM-1 (five studies [46,49,52,64,66] out of 11 [46,49,51,52,57,60,62,64,66,69]) and in P-selectin (three studies [44,62,64] out of six [44,49,60,62,64,69]) after cocoa treatment. In contrast, VCAM-1 decreased in a single trial [60] (out of nine [46,49,52,56,57,60,62,64,69]) and E-selectin did not change in any of six RCTs [49,59,60,62,64,69]. For the interpretation of the results, it is important to consider that E-selectin and VCAM-1 are only expressed by activated endothelial cells, and P-selectin additionally by platelets, whereas ICAM-1 is expressed by a variety of cells: activated endothelial cells, but especially by unstimulated and stimulated leukocytes [85,86]. With regard to the origin of adhesion molecules in serum/plasma, the reduction of ICAM-1 and the lack of changes in VCAM-1 may reflect a reduced activation of leukocytes by cocoa. Nevertheless, caution is necessary when interpreting the results. Changes in serum/plasma may not only reflect changes in the expression of the cell surface, but also changes in the degree of shedding and/or internalization. Moreover, shed molecules may interact with counter receptors or soluble ligands, and thus may not be detected [87]. However, the reduced expression of VLA-4, CD36 (scavenger receptor of oxidized LDL) [64], and CD62 L [59] on monocytes and the decrease in 4-series leukotrienes in plasma after cocoa consumption indicates lowered monocyte activation. This is plausible as leukotriene B_4_ and oxidized LDL, which are known stimuli for monocytes [88], have been shown to decrease through cocoa consumption [43,89]. T cells are involved in atherogenesis [90], but their expression of LFA-1, VLA-4, and CD40 was not modulated by cocoa consumption [64], suggesting that T cells were not modulated. Thus, our results suggest that anti-inflammatory changes after cocoa consumption may be detected by cellular biomarkers (especially on monocytes) rather than by parameters in the serum/plasma. Microparticles derived from endothelium, platelets, and leukocytes induce the transcription and secretion of proinflammatory cytokines and the expression of adhesion molecules and have been considered as biomarkers of vascular injury and inflammation [91]. If we also bear in mind that both studies investigating endothelial microparticles found reduced concentrations in plasma after consumption of flavanol-rich cocoa [59,72], endothelial microparticles are promising parameters for future studies with a focus on vascular inflammation.

With regard to the study quality (Table 6), most trials considered were crossover studies using washout periods, suggesting a well-performed study design. However, allocation concealment and compliance with intervention were not always reported and participants not always blinded. Masking of participants is difficult due to the characteristic color and taste of cocoa, but it is possible, especially through the use of beverages. If we also bear in mind that inflammation was not the primary aim in most studies except for Monagas *et al.* [64], which is the only study with sample size calculation on the basis of inflammatory biomarkers, evidence for anti-inflammatory effects achieved by cocoa consumption is rather low.

## 5. Conclusions

In conclusion, consumption of flavanol-rich cocoa may reduce inflammation, probably by a reduced activation of monocytes and neutrophils. This may prevent or even reduce vascular inflammation. The efficacy of this measure probably depends on the extent of vascular inflammation, but also on the kind of cocoa product used. However, the evidence for anti-inflammatory effects of cocoa consumption is currently low. Further RCTs with inflammation as primary outcome marker are needed. These should investigate specific markers of leukocyte activation not only in the serum or plasma, but also in leukocytes. Endothelial microparticles should also be determined. Subjects suffering from low basal inflammation are of great interest as well as situations accompanied by an increased inflammatory burden (e.g., postprandial state, oral glucose tolerance test). In future trials, researchers and participants should be blinded and compliance with cocoa treatment should be determined to improve the studies’ quality.

## Figures and Tables

**Table 1:** Effect of bolus consumption of cocoa on inflammation—results from randomized, controlled trials.

Study (Reference)	*n*	Participants	Intervention	EC (mg)	Study Design	Sample	Parameter	Results	Annotations
Schramm *et al.*, 2001 [43]	10	Healthy NS Age: 39 ± 1 BMI: 24.0 ± 0.7 CRP: n.d.	I: Flavanol-rich chocolate (Dove dark chocolate, Mars), 37 g, consumed with 45 g bagel	I: 41	Double-blind, crossover	Plasma (0 h, 2 h)	Leukotrienes C_4,_ D_4,_ E_4_ (sum)	I < C (2 h)	Polyphenol-free diet, 1 week washout
			C: Flavanol-low chocolate (Mars), 37 g, consumed with 45 g bagel	C: 2					
Heptinstall *et al.*, 2006 [44]	12	Healthy subjects Age: n.d. (adults) BMI: n.d. CRP: n.d.	I: Cocoa-rich beverages (CocoaPro, Mars) with different flavanol content: I_1_: 980 mg I_2_: 680 mg I_3_: 380 mg	n.d.	Double-blind, crossover	Monocytes from whole blood	CD62P (P-selectin)	I: ↓ C: o	48 h before low-flavonoid diet, ≥10 days washout
							CD11b ^b^	I: ↓ C: ↓	
			C: Cocoa beverage, low in flavanols (80 mg)	n.d.		Neutrophils from whole blood (0 h, 2 h, 4 h, 6 h)	CD62P (P-selectin)	I: ↓ C: o	
							CD11b ^b^	I: ↓ C: ↓	
Flammer *et al.*, 2007 [45]	22	Patients with heart transplantation ^a^ Age: 54 ± 3 BMI: 25.7 ± 0.9 CRP: 5.7 ± 1.9	I: Flavanol-rich dark chocolate (Nestlé Noir Intense, Nestlé), 40 g	I: 36	Double-blind, parallel group	Serum (0 h, 2 h)	CRP ^c^	I: o C: o	
			C: Flavanol-free chocolate (Nestlé), 40 g	C: 0					
Davison *et al.*, 2012 [47]	14	Healthy NS Age: 22 ± 1 BMI: n.d. CRP: n.d.	I: Flavanol-rich dark chocolate (Nestlé Noir Intense, Nestlé), 100 g	I: 97	Crossover	Plasma	IL-6	I: o C_1_: o C_2_: o	Cycling for 2.5 h at 60% maximum O_2_ uptake
						Neutrophils from whole blood (0 h up to 1 h post-exercise)	Respiratory burst ^d^	I: o C_1_: o C_2_: o	
							Degranulation ^e^	I: o C_1_: o C_2_: o	
			C_1_: Cocoa-free chocolate, 71 g	C_1_: 0					
			C_2_: No cocoa product	C_2_: 0					
Flammer *et al.*, 2012 [48]	20	Congestive heart failure, NS Age: 59 ± 3 BMI: 25.8 ± 1.0 CRP: 2.9 ± 0.7	I: Flavanol-rich dark chocolate (Nestlé Noir Intense, Nestlé), 40 g	I: 36	Double-blind, parallel group	Plasma (0 h, 2 h)	CRP ^c^	I: o C: o	24 h before low-flavonoid diet
			C: Cocoa-liquor-free chocolate (Nestlé), 28.4 g, weight-matched for fat and sugar content	C: 0					
Mellor *et al.*, 2013 [49]	10	Type 2 diabetes, stable, treated with metformin or lifestyle Age: 61 (42–68) ^f^ BMI: 32.5 ± 6.0 CRP: n.d.	I: Flavanol-rich chocolate (Acticoa, Barry Callebaut), 13.5 g, 1 h before an oral glucose load	n.d.	Placebo-controlled, double-blind, crossover	Serum (0 h and 2 h after an oral 75-g-glucose load)	ICAM-1	I: o C: ↑ ΔI < ΔC	2 weeks run-in without polyphenol-rich foods
				n.d.			E-selectin	I: o C: o ΔI < ΔC	
			C: Low-flavanol chocolate (Barry Callebaut), 13.5 g, 1 h before an oral glucose load				P-selectin	I: o C: o ΔI = ΔC	
							P-selectin-glyco-protein ligand 1	I: o C: o ΔI < ΔC	
Vázquez-Agell *et al.*, 2013 [46]	18	Healthy NS Age: 26 ± 7 BMI: n.d. CRP: n.d.	I: Cocoa-rich beverages (Nutrexpa) I_1_: with 250 mL milk I_2_: with 250 mL water	I_1_: 28 I_2_: 28	Crossover	Serum (0 h, 6 h)	E-selectin	I_1_: o I_2_: ↓ C: o	7 days cocoa-free run-in, 48 h before low-polyphenol-diet
							ICAM-1	I_1_: ↓ I_2_: ↓ C: o I_2_ < I_1_ (6 h)	
			C: Pure milk, 250 mL	C: 0			VCAM-1	I_1_: o I_2_: o C: o	
						Peripheral blood mononuclear cells (0 h, 6 h)	P-p65/β-actin	I_1_: o I_2_: ↓ C: ↑ I_2_ < I_1_ (6 h)	
Loffredo *et al.*, 2014 [71]	20	Peripheral artery disease ^g^ Age: 69 ± 2 BMI: 27 ± 1 CRP: n.d.	I: Dark chocolate (≥85% cocoa), 40 g/day C: Milk chocolate (≤35% cocoa), 40 g/day	unclear	Crossover, single-blind	Serum (0 h, 2 h)	NOX2-derived peptide	I: ↓ C: o	
Basu *et al.*, 2015 [50]	18	Type 2 diabetes, stable ≥5 years, no insulin, obese, NS Age: 56 ± 3 BMI: 35.3 ± 2.0 CRP: 5.3 ± 1.2	I: Cocoa drink, provided in closed lip cups, consumed with a high-fat-fast-food-style breakfast	I: 40	Double-blind, crossover	Serum (0 h, 0.5 h, 1 h, 2 h, 4 h, 6 h)	CRP ^c^	I: o C: o ΔI = ΔC	24 h before no polyphenol-rich food, 3 days food records
			C: Cocoa-free placebo drink, provided in closed lip cups, consumed with a high-fat-fast-food-style breakfast	C: 0					

^a^ 41% Former smokers, 27% dyslipidemia, 32% hypertension, 18% diabetes, 41% former myocardial infarction or peripheral artery disease; ^b^ collagen-induced activation of cells in whole blood *ex vivo*; ^c^ determined by high-sensitivity test kits for C-reactive protein; ^d^ stimulation with bacterial extract; ^e^ measured by elastase; ^f^ median and interquartile range in parentheses; ^g^ 80% former smokers, 90% dyslipidemia, 85% hypertension, 30% diabetes, 40% coronary heart disease; BMI: body mass index; C: control; CRP: C-reactive protein; EC: epicatechin intake; I: intervention; ICAM-1: intercellular adhesion molecule-1; IL-6: interleukin-6; n.d.: no data available; NS: nonsmoker; VCAM-1: vascular cell adhesion molecule-1; ↑: increase; ↓: decrease;o: no changes, Δ changes. Data on age (years), BMI (kg/m^2^), and CRP (mg/L) are means ± SEM if not indicated otherwise. Means were calculated as weighted means from the data of individual groups if not provided by the authors. Missing SEMs were calculated by SDs of individual groups. *n* refers to the number of participants for whom data on inflammatory markers were available.

**Table 2:** Effect of regular cocoa consumption on inflammation in healthy subjects—results from randomized, controlled trials.

Study (Reference)	*n*	Participants	Intervention	EC (mg)	IP (d)	Study Design	Sample	Parameter	Results	Annotations
Grassi *et al.*, 2005 [51]	20	Healthy NS Age: 34 ± 2 BMI: 22.6 ± 0.6 CRP: 0.3 ± 0.1	I: Dark chocolate (Ritter Sport, Halbbitter, Ritter), 100 g/day	I: 66	15	Crossover	Serum	CRP ^a^	I: o C: o	No flavonoid-rich food, food diaries, 1 week run-in and 1 week washout without chocolate
			C: White chocolate (Milka, Kraft Foods), 90 g/day	C: 0				ICAM-1	I: o C: o	
Kurlandsky and Stote, 2006 [52]	24	Healthy NS, serum cholesterol 4.1–7.8 mmol/L, no lipid lowering medication Age: 43 ± 2 BMI: 25.0 ± 0.8 CRP: 4.7 ± 1.5	I: Dark chocolate (Dove Silky Dark Chocolate, Mars), 41 g/day	n.d.	42	Parallel group	Serum	CRP ^a^	I: o C: o	Flavonoid-rich food limited, 3 days food records before and in week 2, 4, and 6 ofintervention
			C: No chocolate					ICAM-1	I: ↓ C: o	
								VCAM-1	I: o C: o	
Crews *et al.*, 2008 [53]	88	Healthy subjects with prehypertension Age: 69 ± 1 BMI: 25.3 ± 0.5 CRP: 1.6 ± 0.2	I: Dark chocolate, 37 g/day, + cocoa beverage, 237 mL/day (Hershey)	n.d.	42	Placebo-controlled, double-blind, parallel group	Serum	CRP ^a^	ΔI = ΔC	No flavonoid-rich food, 1 week run-in without flavonoid-rich food, compliance: daily records
			C: Cocoa-free placebo products (Hershey)	n.d.						
Njike *et al.*, 2011 [54]	44	Healthy NS, overweight/obese Age: 52 ± 2 BMI: 30.2 ± 3.4 CRP: 0.9 (median)	I: Cocoa-rich beverage with water (Hershey), 454 g/day I_1_: sugared I_2_: sugar- free (I_2_)	I_1_: 48 I_2_: 48	42	Double-blind, crossover	Serum	CRP ^a^	I_1_: o I_2_: o C: o	No flavonoid-rich food 24 h before, 3 days food diary during each treatment, 4 weeks washout
			C: Cocoa-free sugared placebo beverage (Hershey), 454 g/day	C: 0						
Tzounis *et al.*, 2011 [55]	22	Healthy subjects Age: 30 ± 3 BMI: 23.2 ± 0.4 CRP: 0.3 ± 0.1	I: Flavanol-rich cocoa drink (Mars), 150 mL/day	I: 89	28	Placebo-controlled, double-blind, crossover	Plasma	CRP ^a^	I: ↓ C: o	2 weeks run-in, 4 weeks washout, no flavonoid-rich food, 4 days food diaries during run-in, 3rd week of both treatments, and during 4th week washout compliance: % used cocoa sachets, self-reported intake >95%
			C: Low-flavanol cocoa drink (Mars), 150 mL/day	C: 3						
Ibero-Baraibar *et al.*, 2014 [56]	50	Healthy NS, overweight/obese Age: 57 ± 1 BMI: 30.6 ± 0.3 CRP: n.d.	I: Cocoa extract (1.4 g/day; Nutrafur) in ready-to-eat meals, within a hypocaloric diet (energy restriction of 15%)	I: 153	28	Double-blind, parallel group	Plasma	ICAM-1	I: ↓ C: ↓	1 week run-in without cocoa, 3 days before low-polyphenol-diet; exclude polyphenol-rich foods; compliance >98%
			C: Ready-to-eat meals not enriched with cocoa extract, within a hypocaloric diet (energy restriction of 15%)	C: 0				VCAM-1	I: o C: o	
Sarriá *et al.*, 2014 [57]	44	Healthy NS, normocholesterolemic (*n* = 24; N) or moderately hypercholesterolemic (*n* = 20; H) Age: 29 ± 1 BMI: 23.6 ± 0.5 CRP: n.d.	I: Cocoa drink with cocoa, 30 g/day, rich in dietary fiber (Nutrexpa), with 400 mL semi-skimmed milk	I: 9.3	28	Crossover	Plasma	CRP	n.d.	2 weeks run-in and during intervention, polyphenol-rich foods were restricted, 3-day food records, compliance measured
								IL-1β	I: o C: o (N, H)	
								IL-6	I: o C: o (N, H)	
			C: Cocoa-free drink with semi-skimmed milk, 400 mL/day	C: 0				IL-8	I: o C: o (N, H)	
								IL-10	I: ↓ C: ↓ I_p_ < C_p_ (N, H)	
								TNF-α	I: o C: o (N, H)	
								MCP-1	I: o C: o (N, H)	
								VCAM-1	I: o C: o (N, H)	
								ICAM-1	I: o C: o (N, H)	
West *et al.*, 2014 [58]	13	Healthy NS, Overweight/obese Age: 52 ± 0.3 BMI: 27.8 ± 0.2 CRP: n.d.	I: Dark chocolate, 37 g/day + cocoa drink with 22 g cocoa/day	I: 73.6	42	Placebo-controlled, crossover	Plasma	CRP ^a^	n.d.	2 days before no flavonoid-rich foods, 2 weeks washout
			C: Low-flavanol chocolate + cocoa-free drink as color matched control					IL-1β	I: o C: o	
				C: 0.9				IL-6	I: o C: o	
								TNF-α	I: o C: o	
McFarlin *et al.*, 2015 [59]	24	Healthy subjects with normal weight (*n* = 10; N), overweight (*n* = 7; O), or obesity (*n* = 7; B), Age: 22 ± 2 BMI: 21.6 ±1.9 (N), 27.0 ± 1.4 (O), 34.9 ± 9.9 (B) CRP: n.d.	I: Cocoa bars with 12.7 g natural cocoa (Hershey) C: Cocoa-free placebo bar (Hershey), matched for energy content and macronutrient composition	I: 48.0 C: 0	42	Placebo-controlled, crossover	Serum/Plasma	CRP ^a^	n.d.	no chocolate during study, 2 weeks washout
								AGP	n.d.	
								AMG	n.d.	
								Adipsin	n.d.	
								Haptoglobin	I: o (N,O), ↓ (B) C: o (N, O, B) t_0_: B > O > N	
								E-selectin	I: o (N, O, B) C: o (N, O, B) t_0_: B > O, N	
								SAP	n.d.	
								Endocan-1	I: o C: o (N, O, B)	
								IL-1β	n.d.	
								IL-6	n.d.	
								IL-8	n.d.	
								TNF-α	n.d.	
							Non-inflammatory monocytes (CD16-)	CD11b	n.d.	
							Proinflammatory monocytes (CD16+)	CD11b	n.d.	
							All Monocytes	CD62L	I: ↑ (N) o (O), ↓ (B) C: o (N, O, B) t_0_: B > O > N	
							Proinflammatory monocytes (CD16+)	CD62L	I: ↑ (N, O), ↓ (B) C: o (N, O, B) t_0_: B > O > N	
							EMP in whole blood	EMP concentration	I: o (N, O), ↓ (B) C: o (N, O, B) t_0_: B > O > N	

^a^ determined by high-sensitivity test kits for C-reactive protein. AGP: alpha-2 acid glycoprotein; AMP: alpha-2 macroglobulin; B: obese; BMI: body mass index; C: control; C_p_: value after intervention; d: days; CRP: C-reactive protein; EC: epicatechin; EMP: endothelial microparticles (CD42a-/45-/144+); H: hypercholesterolemic; I: intervention; IP: intervention period; IL: interleukin; I_p_: value after intervention; ICAM-1: intracellular adhesion molecule 1; IL-1β: interleukin-1β; IL-6: interleukin-6; n.d; no data available; NS: non-smoker; MCP-1: monocyte chemoattractant protein-1; N: normocholesterolemic; O: overweight; SAP: serum amyloid P; TNF-α: tumor necrosis factor-α; VCAM-1: vascular cell adhesion molecule-1; wk: week; ↑: increase; ↓: decrease; o: no changes, Δ: difference pre- *vs.* post-consumption value. Data on age (years), BMI (kg/m^2^), and CRP (mg/L) are means ± SEM if not indicated otherwise. Means were calculated as weighted means from the data of individual groups if not provided by the authors. Missing SEMs were calculated by SDs of individual groups. *n* refers to the number of participants for which data on inflammatory markers were available.

**Table 3:** Effect of regular cocoa consumption on inflammation in patients with pre-/hypertension—results from randomized, controlled trials.

Study (Reference)	*n*	Participants	Intervention	EC (mg)	IP (d)	Study Design	Sample	Parameter	Results	Annotations
Grassi *et al.*, 2005 [51]	20	Untreated grade I hypertension, NS Age: 44 ± 2 BMI: 25.4 ± 0.3 CRP: 0.4 ± 0.1	I: Flavanol-rich dark chocolate (Ritter Sport Halbbitter, Ritter), 100 g/day	I: 66	15	Crossover	Serum	CRP ^a^	I: o C: o	No flavonoid-rich food, Food diaries, 1 week chocolate free run-in and washout
								ICAM-1	I: o C: o	
			C: White chocolate (Milka, Kraft Foods), 90 g/day, matched for energy, macro-, micronutrients	C: 0						
Wang-Polagruto *et al.*, 2006 [60]	32	Hypertension stage 1 or prehypertension, hypercholesterolemia, NS Age: 57 ± 1 BMI: 25.1 ± 0.6 CRP: n.d.	I: Flavanol-rich cocoa drink with 18.8 g cocoa powder (Mars), sucrose, 240 mL/day	n.d.	42	Double-blind, parallel group	Plasma	P-selectin	I: o C: o	No flavonoid-rich food 24 h before study, 2 weeks run-in with flavanol-poor cocoa drink, 3 × 3 days Food records, Compliance: empty packets
								E-selectin	I: o C: o	
			C:_Flavanol-poor cocoa drink, 240 mL/day	n.d.				ICAM-1	I: o C: o	
								VCAM-1	I: ↓ C: o ΔI > ΔC	
Grassi *et al.*, 2008 [61]	19	Untreated stage I hypertension, impaired glucose tolerance, NS Age: 45 ± 1 BMI: 26.5 ± 0.3 CRP: n.d.	I: Flavanol-rich chocolate (Cuorenero, Sugar Company), 100 g/day	I: 111	15	Crossover	Serum	CRP ^a^	I: o C: o	No flavonoid-rich food, 1 week cocoa-free run-in and washout, Food records daily
			C: White chocolate (Milka, Kraft Foods), 100 g/day	C: 0						
Muniyappa *et al.*, 2008 [62]	20	Stage 1 hypertension, overweight/obesity, NS, antihypertensive medication (*n* = 6) discontinued Age: 51 ± 2 BMI: 33.2 ± 1.4 CRP: n.d.	I: Flavanol-rich cocoa drink with cocoa powder (CocoaPro, Mars), water, 300 mL/day	I: 174	14	Placebo-controlled, double-blind, crossover	Serum or plasma	E-selectin	I: o C: o	1 week Run-in and 1 week washout with low-flavanol diet, Compliance: cocoa powder or placebo packets
								ICAM-1	I: o C: o	
								VCAM-1	I: o C: o	
			C: Flavanol-poor placebo drink, with water, matched for energy, macronutrients, similar in color, taste and packaging, 300 mL/day	C: 2				MCP-1	I: o C: o	
								IL-6	I: o C: o	
								TNF-α	I: o C: o	

^a^ determined by high-sensitivity test kits for C-reactive protein. BMI: body mass index; C: control; d: days; CRP: C-reactive protein; EC: epicatechin; I: cocoa intervention; ICAM-1: intercellular adhesion molecule-1; I: cocoa intervention; IL-6: interleukin-6; IP: intervention period; n.d.: no data available; MCP-1: monocyte chemoattractant protein-1; TNF-α: tumor necrosis factor-α; VCAM-1: vascular cell adhesion molecule-1; ↓: decrease; o: no changes; ∆ difference pre- *vs.* post-consumption values. Data on age (years), BMI (kg/m^2^), and CRP (mg/L) are means ± SEM. Means were calculated as weighted means from the data of individual groups if not provided by the authors. Missing SEMs were calculated by SDs, of individual groups. *n* refers to the number of participants for whom data on inflammatory markers were available.

**Table 4:** Effect of regular cocoa consumption on inflammation in patients with type 2 diabetes or impaired glucose tolerance—results from randomized, controlled trials.

Study (Reference)	*n*	Participants	Intervention	EC (mg)	IP (d)	Study Design	Sample	Parameter	Results	Annotations
Balzer *et al.*, 2008 [63]	41	Type 2 diabetes, stably-treated, NS Age: 64 ± 1 BMI: 31.6 ± 0.8 CRP: 4.9 ± 1.1	I: Flavanol-rich cocoa drink, with CocoaPro cocoa powder (Mars), 750 mL/day	I: 203	30	Double-blind, parallel group	Plasma	CRP	I: o C: o	No dietary restrictions, dietary intake not determined, compliance: empty cocoa sachets, epicatechin in plasma
			C: Flavanol-poor cocoa drink with CocoaPro cocoa powder (Mars), similar in taste, matched for energy and macro-, micronutrient composition, 750 mL/day	C: 17						
Grassi *et al.*, 2008 [61]	19	Impaired glucose tolerance, untreated stage I hypertension, NS Age: 45 ± 1 BMI: 26.5 ± 0.3 CRP: n.d.	I: Flavanol-rich chocolate (Cuorenero, Sugar Company), 100 g/day	I: 111	15	Crossover	Serum	CRP ^a^	I: o C: o	No flavonoid-rich food, 1 week cocoa-free run-in and washout, food records daily
			C: White chocolate (Milka, Kraft Foods), 100 g/day	C: 0						
Monagas *et al.*, 2009 [64]	42	Diabetes or ≥3 cardiovascular disease risk factors (smoking, hypertension, hypercholesterolemia, obesity, family history of premature coronary heart disease) Age: 70 ± 2 BMI: 27.6 ± 0.8 CRP: 0.5 ± 0.3	I: Cocoa drink, prepared with cocoa powder (Nutrexpa) and skim milk, 500 mL/day	I: 46	28	Crossover	Serum or plasma	CRP ^a^	I: o C: o	Flavonoid-rich food limited, 3 × 3 days food records, ompliance: patient reports and epicatechin metabolites in plasma and urine, 2 weeks run-in, no washout-period
			C: Skim milk, 500 mL/day	C: 0				P-selectin	I: ↓ C: o I < C (d28)	
								E-selectin	I: o C: o	
								ICAM-1	I: ↓ C: o I < C (d28)	
								VCAM-1	I: o C: o	
								MCP-1	I: o C: o	
								IL-6	I: o C: o	
							Monocytes	LFA-1	I: o C: o	
								Mac-1	I: o C: o	
								VLA-4	I: ↓ C: o I < C (d28)	
								SLe^x^	I: o C: o	
								CD36	I: ↓ C: o I < C (d28)	
								CD40	I: ↓ C: o I < C (d28)	
							T cells	LFA-1	I: o C: o	
								VLA-4	I: o C: o	
								SLe^x^	I: o C: o	
								CD40	I: o C: o	
Mellor *et al.*, 2010 [65]	12	Type 2 diabetes, no steroids, no changes in chronic medication Age: 68 (median); 42–71 (range) BMI: n.d. CRP: 2.8 ± 0.5	I: Polyphenol-rich chocolate (Nestlé), 45 g/day	I: 17	56	Double-blind, crossover	Serum or plasma	CRP ^a^	I: o C: o	4 weeks washout, compliance: empty wrappers: 93.8%, no changes in diet and lifestyle, dietary recalls
			C: Polyphenol-free chocolate (Nestlé), 45 g/day, matched for energy and macronutrients	C: <2						
Stote *et al.*, 2012 [66]	19	Impaired or normal glucose tolerance, obese, NS Age: 46 ± 1 BMI: 36.8 ± 0.2 CRP: n.d.	I: Cocoa drink with 28 g cocoa (different flavanol content, Mars), water, 300 mL/day	I_1:_ 184 I_2_: 72 I_3_: 34	5	Single-blind, crossover	Serum or plasma (0, 0.5, 1, 1.5, 2.0 h after an oral 75-g-glucose load)	CRP ^a^	↓ by dose	10 days washout, no dietary changes
								ICAM-1	o by dose	
								IL-6	↓ by dose	
			C: Flavanol-poor drink with water (Mars), 300 mL/day, matched for energy, macro-, micronutrients	C: 4						
Parsaeyan *et al.*, 2014 [67]	100	Type 2 diabetes Age: 54 ± 1 BMI: 28 ± 0.5 CRP: n.d.	I:_Cocoa drink (10 g cocoa, 10 g milk powder, 250 mL water), 2 drinks/day	n.d.	42	Parallel group	Serum	CRP^a^	I: ↓ C: o	Dietary records, no restrictions
								IL-6	I: ↓ C: o	
								TNF-α	I: ↓ C: o	
			C: Cocoa-free drink (10 g milk powder, 250 mL water), 2 drinks/day	n.d.						
Rostami *et al.*, 2015 [68]	60	Type 2 diabetes, blood pressure ≥140/90 mm Hg, stable medication, NS Age: 60 ± 1 BMI: 29.8 ± 0.6 CRP: unclear	I: Dark chocolate (Farmand), 25 g/day	n.d.	56	Placebo-controlled, double-blind, parallel group	Serum	CRP ^a^	I: ↓ C: o; No effect by ANCOVA adjusted for age, sex, energy intake	No further chocolate, 3 days food records
			C: White chocolate (Farmand), 25 g/day, isocaloric, same color and shape, identically wrapped	n.d.						

^a^ determined by high-sensitivity test kits for C-reactive protein. BMI: body mass index; C: control; d: days; CRP: C-reactive protein; EC: epicatechin; I: cocoa intervention; ICAM-1: intercellular adhesion molecule-1; IL-6: interleukin-6; IP: intervention period; LFA-1: lymphocyte function-associated antigen-1; MCP-1: monocyte chemoattractant protein; n.d.: no data available; SLe^x^: sialil Lewis X, CD15s; VCAM-1: vascular cell adhesion molecule-1; VLA-4: very late activation antigen-4; ↓: decrease; o: no changes; Δ difference pre- *vs.* post-consumption values. Data on age (years), BMI (kg/m^2^), and CRP (mg/L) are means ± SEM if not indicated otherwise. Means were calculated as weighted means from the data of individual groups if not provided by the authors. Missing SEMs were calculated by SDs, of individual groups. *n* refers to the number of participants for whom data on inflammatory markers were available.

**Table 5:** Effect of regular cocoa consumption on inflammation in patients with coronary heart disease—results from randomized, controlled trials.

Study (Reference)	*n*	Participants	Intervention	EC (mg)	IP (d)	Study Design	Sample	Parameter	Results	Annotations
Farouque *et al.*, 2006 [69]	40	Coronary artery disease, 55% with hypertension, 95% with hypercholesterolemia, mostly medically treated Age: 61 ± 6 BMI: 27.5 ± 2.4 CRP: 1.2 (median)	I: Flavanol-rich chocolate (48 g/day; Mars) + one cocoa beverage per day (Mars)	I: 107	42	Placebo-controlled, double-blind, crossover	Plasma	CRP ^a^	I: o C: o	No dietary restrictions, dietary intake not determined, compliance: wrapper amount and patient reports
								P-selectin	I: o C: o	
			C: Isocaloric placebos	C: 5				E-selectin	I: o C: o	
								ICAM-1	I: o C: o	
								VCAM-1	I: o C: o I > C (d0) I = C (d42)	
Heiss *et al.*, 2010 [70]	16	Coronary artery disease, medically treated, NS Age: 64 ± 3 BMI: 27.8 ± 1.8 CRP: 1.8 ± 0.4	I: Flavanol-rich cocoa drink with CocoaPro cocoa powder (Mars), 2 drinks/day, prepared with skim milk or water	I: 59	30	Double-blind, crossover	Plasma	CRP ^a^	I: o C: o	1 week washout
							Peripheral blood mononuclear cells	Chemotaxis	I: o C: o	
			C: Flavanol-poor cocoa drink, 2 drinks/day, with skim milk or water, matched for energy, macro-, micronutrients; similar in taste and package	C: 1						
Flammer *et al.*, 2012 [48]	20	Congestive heart failure, NS Age: 59 ± 3 BMI: 25.8 ± 1.0 CRP: 2.9 ± 0.7	I: Flavanol-rich dark chocolate (Nestlé Noir Intense, Nestlé), 40 g/day	I: 36	28	Double-blind, parallel group	Plasma	CRP ^a^	I: o C: o	24 h before flavonoid-low diet
			C: Cocoa-liquor-free chocolate (Nestlé), 28.4 g/day, matched for fat and sugar content, identically wrapped	C: 0						
Horn *et al.*, 2014 [72]	16	Coronary artery disease, NS, medically treated, 38% diabetes, 88% hypertension, 96% hyperlipidemia, 63% prior smoking Age: 64 ± 1 BMI: 28.8 ± 0.5 CRP: 1.8 ± 0.1	I: Flavanol-rich cocoa drink with cocoa powder (Mars), 2 drinks/day	I: 118	30	Double-blind, crossover	Plasma	EMP (CD41+)	I: o C: o	1 week washout
			C: Flavanol-poor cocoa drink with cocoa powder (Mars), 2 drinks/day, matched for macro- and micronutrients, energy, and methylxanthines	C: 2				EMP (CD144+)	I: ↓ C: o I < C (d30)	
								EMP (CD31+/41-)	I: ↓ C: o I < C (d30)	

^a^ determined by high-sensitivity test kits for C-reactive protein. BMI: body mass index; C: control; d: days; CRP: C-reactive protein; EC: epicatechin; EMP: endothelial microparticles; I: cocoa intervention; ICAM-1: intercellular adhesion molecule-1; IL-6: interleukin-6; IP: intervention period; n.d.: no data available; PMP: platelet-derived microparticles; VCAM-1: vascular cell adhesion molecule-1; ↓: decrease; o: no changes; Δ difference pre- *vs.* post-consumption values. Data on age (years), BMI (kg/m^2^), and CRP (mg/L) are means ± SEM if not indicated otherwise. Means were calculated as weighted means from the data of individual groups if not provided by the authors. Missing SEMs were calculated by SDs, of individual groups. *n* refers to the number of participants for whom data on inflammatory markers were available.

**Table 6:** Quality of randomized controlled studies.

Study (Reference)	Allocation Concealment	Masking of Participants	Masking of Researchers	Dropouts Clearly Reported	Industry Funding	Cocoa Products as Gift from Industry	Compliance Measured	Crossover Design	Carry-Over Effects Possible ^a^	Dietary Intake Documented	Dietary Restrictions
Bolus consumption											
Schramm *et al.*, 2001 [43]	Y	Y	Y	Y	N	Y	-	Y	N	-	Y
Heptinstall *et al.*, 2006 [44]	?	Y	Y	-	N	Y	-	Y	N	-	Y
Flammer *et al.*, 2007 [45]	Y	Y	Y	-	N	Y	-	N	-	-	Y
Davison *et al.*, 2012 [47]	Y	N	Y	-	Y	Y	-	Y	N	Y	Y
Flammer *et al.*, 2012 [48]	Y	Y	Y	Y	Y	Y	-	N	-	-	Y
Mellor *et al.*, 2013 [49]	Y	Y	Y	-	Y	Y	-	Y	N	-	Y
Vázquez-Agell *et al.*, 2013 [46]	?	N	N	Y	N	Y	-	Y	N	-	Y
Loffredo *et al.*, 2014	Y	N	Y	-	N	N	-	Y	N	-	N
Basu *et al.*, 2015 [50]	?	Y	Y	-	N	N	-	Y	N	-	Y
Regular consumption—healthy											
Grassi *et al.*, 2005 [51]	?	N	N	Y	N	?	N	Y	N	Y	Y
Kurlandsky and Stote, 2006 [ 52]	Y	N	N	Y	N	Y	N	N	-	Y	Y
Crews *et al.*, 2008 [53]	Y	Y	Y	Y	Y	Y	Y	N	-	N	Y
Njike *et al.*, 2011 [54]	Y	Y	Y	Y	Y	Y	N	Y	N	Y	Y
Tzounis *et al.*, 2011 [55]	Y	Y	Y	Y	N	Y	Y	Y	N	Y	Y
Ibero-Baraibar *et al.*, 2014 [56]	?	Y	Y	N	N	N	Y	N	-	Y	Y
Sarriá *et al.*, 2014 [57]	N	N	N	Y	Y	Y	Y	Y	Y	Y	Y
West *et al.*, 2014 [58]	?	Y	Y	Y	Y	Y	N	Y	N	N	Y
McFarlin *et al.*, 2015 [59]	?	Y	Y	N	Y	Y	N	Y	N	N	Y
Regular consumption—pre-/hypertension											
Grassi *et al.*, 2005 [51]	?	N	N	Y	N	?	N	Y	N	Y	Y
Wang-Polagruto *et al.*, 2006 [60]	?	Y	Y	Y	Y	Y	Y	N	-	Y	Y
Grassi *et al.*, 2008 [61]	?	N	Y	Y	N	Y	N	Y	N	Y	Y
Muniyappa *et al.*, 2008 [62]	Y	Y	Y	Y	N	Y	Y	Y	N	N	Y
Regular consumption—diabetes, impaired glucose tolerance											
Balzer *et al.*, 2008 [63]	?	Y	Y	Y	N	Y	Y	N	-	N	N
Grassi *et al.*, 2008 [61]	?	N	Y	Y	N	Y	N	Y	N	Y	Y
Monagas *et al.*, 2009 [64]	?	N	Y	Y	N	Y	Y	Y	Y	Y	Y
Mellor *et al.*, 2010 [65]	Y	Y	Y	Y	N	Y	Y	Y	N	Y	N
Stote *et al.*, 2012 [66]	?	N	Y	Y	N	Y	N	Y	N	N	Y
Parsaeyan *et al.*, 2014 [67]	N	N	N	-	N	N	N	N	-	Y	N
Rostami *et al.*, 2015 [68]	Y	N	Y	Y	N	Y	Y	N	-	Y	N
Regular consumption—coronary heart disease											
Farouque *et al.*, 2006 [69]	Y	Y	Y	N	Y	Y	Y	Y	Y	N	N
Heiss *et al.*, 2010 [70]	?	Y	Y	N	Y	Y	N	Y	N	N	N
Flammer *et al.*, 2012 [48]	Y	Y	Y	Y	Y	Y	Y	N	-	N	Y
Horn *et al.*, 2014 [72]	Y	Y	Y	-	Y	Y	N	Y	N	N	N

^a^ concerns studies with crossover design: In case of washout periods were reported, carry-over effects could be excluded, but not if washout periods were missing or not reported. All criteria except for “dietary intake documented” and “dietary restrictions” are GRADE criteria. N: no, Y: yes; ?: unclear.
